# High-Efficiency Fiber Edge Coupling for Silicon Nitride Integrated Photonics

**DOI:** 10.3390/mi16121401

**Published:** 2025-12-12

**Authors:** Sergey S. Avdeev, Aleksandr S. Baburin, Evgeniy V. Sergeev, Alexei B. Kramarenko, Arseniy V. Belyaev, Danil V. Kushnev, Kirill A. Buzaverov, Ilya A. Stepanov, Vladimir V. Echeistov, Ales S. Loginov, Sergey V. Bukatin, Ali Sh. Amiraslanov, Evgeniy S. Lotkov, Dmitriy A. Baklykov, Ilya A. Rodionov

**Affiliations:** 1 Shukhov Labs, Quantum Park, Bauman Moscow State Technical University, 105005 Moscow, Russia; avdeevss@bmstu.ru (S.S.A.); baburin@bmstu.ru (A.S.B.); sergeev_e@bmstu.ru (E.V.S.); kramarenko@bmstu.ru (A.B.K.); arsbel99@gmail.com (A.V.B.); dvkushnev@gmail.com (D.V.K.); kirillbuz@bmstu.ru (K.A.B.); stepanovia@bmstu.ru (I.A.S.); wecheistov@bmstu.ru (V.V.E.); loginov@bmstu.ru (A.S.L.); bukatin@bmstu.ru (S.V.B.); amiraslanov@bmstu.ru (A.S.A.); lotevg@bmstu.ru (E.S.L.); dimabaklykov@bmstu.ru (D.A.B.); 2 Dukhov Automatics Research Institute (VNIIA), 127055 Moscow, Russia

**Keywords:** integrated photonics, edge coupling, coupling efficiency

## Abstract

Photonic integrated circuits play a crucial role in almost every aspect of modern life, such as data storage, telecommunications, medical diagnostics, green energy, autonomous driving, agriculture, and high-performance computing. To fully harness their benefits, an efficient coupling mechanism is required to successfully launch light into on-chip waveguides from fibers. This study introduces low-loss coupling strategies and their implementation for silicon nitride integrated photonics. Here we present an overview of coupling technologies, optimized designs, and a fabrication technique for inverse tapers, which enable effective coupling for both transverse-magnetic and transverse-electric modes. We measured the coupling losses of 0.15 dB for UHNA-7 fiber at 1550 nm per facet for single-mode 220 × 1200 nm waveguides. We also designed, fabricated, and experimentally characterized a multi-tip taper, yielding 1.5 dB per facet at 1550 nm with broadband stability over 1500–1600 nm. We believe that our approach is universal and can be used both for individual fiber and fiber arrays coupling and for subsequent assembly of fiber with a chip, ensuring minimal losses.

## 1. Introduction

With the growing demand for high-speed and compact devices, photonic integrated circuits (PICs) are attracting significant interest because of their high bandwidth and compatibility with large-scale integration technologies. Silicon nitride (Si_3_N_4_) is the ideal platform for photonic integrated circuit applications such as Li-DAR [1], optical sensing [2,3,4,5,6,7], bio-spectroscopy [8], communication [9], and for uses in the quantum domain, such as quantum key distribution (QKD), quantum computing [10,11], and quantum sensing [12]. In recent years, Si_3_N_4_ has attracted significant attention as a PIC platform because of its low propagation loss, compatibility with heterogeneous integration, extended transparent bandwidth, and lower susceptibility to errors during the lithography and etching processes compared with the silicon-on-insulator (SOI) platform [13,14,15,16]. Typically, PICs require an effective coupling mechanism to launch light into the waveguide from a fiber. For this purpose, grating and edge couplers are widely studied [17,18,19,20,21,22,23,24,25,26,27,28,29,30,31,32,33,34,35,36,37,38,39,40,41,42,43]. Grating couplers are used in out-of-plane coupling. For high-volume manufacturing by standard production techniques, for example, in Multi-Project Wafer (MPW) fabs, gratings exhibit a rather low coupling efficiency of ∼5.0 dB/grating coupler and inherently suffer from limited wavelength bandwidth [17,18,19,20,21,22,23]. To enhance the coupling efficiency, an inverse taper could be introduced, as it demonstrates a coupling loss down to values lower than 0.5 dB [23,24,25,26]. However, it should be noted that waveguide type and dimensions and fiber/waveguide mode size differences play a crucial role in coupling efficiency.

Edge couplers exhibit high in-plane light coupling efficiency and a broader spectral bandwidth. Usually, edge couplers are designed to entail long adiabatic tapers with lengths, sometimes exceeding several hundred micrometers, thereby enhancing the mode transfer efficiency. Several techniques make it possible to further improve coupling efficiency, like the 2 × 2 couplers [29,30,31,32,33], mode multiplexers [20,33,34,35], and polarization splitters/rotators [36,37,38,39,40,41,42,43]. The compact form of the adiabatic taper drastically facilitates its applicability in high-density PICs [17]. To provide low-loss edge coupling, special waveguide structures, which serve as mode size converters, are used [44,45]. The optical mode size and shape change during propagation through the tapered waveguide to achieve higher coupling efficiency between two modes with different cross-sections. They are designed to operate adiabatically: the waveguide local first-order mode should propagate through the tapered waveguide while undergoing relatively little mode conversion compared with the higher-order modes or radiation modes. This adiabatic operation is realized in the taper design by gradually increasing the taper cross-section size and decreasing the mode size from the typical diameter of 5–10 μm in fiber to the order of several microns in the waveguide. Different designs of adiabatic tapers have been proposed for Si_3_N_4_ platforms, including linear [46], exponential [22], parabolic [47], and multi-sectional tapers [24,25]. Linear adiabatic tapers are widely used on Si_3_N_4_ and SOI platforms due to their simplicity and robust performance [23,24,31,44,47]. In this linear adiabatic taper mode, conversion occurs more easily in the wider portion [22]. To obtain a short and efficient taper, various taper configurations including nonlinear, stepwise cascaded, multilayered, and metamaterials, as well as double-tip and sinusoidal taper, were extensively analyzed for various photonic platforms [21,22,23,24,25,26,27,28,29]. The main published results for Si_3_N_4_ platform low-loss edge couplers are presented in Table 1.

Currently, the best achieved efficiency of 0.17 dB was demonstrated by the adiabatic Si_3_N_4_ coupler based on a taper with a length of 500 μm for UHNA-3 fiber/100 × 900 nm waveguide interface, cleaved with a good cleaving position tolerance [37] and, in a recent publication by PSI Quantum, reporting 0.052 ± 0.012 dB losses with UHNA-4 fiber [49]. However, as this work is published by a commercial company, details on taper design, device architecture and fabrication process are not disclosed. Moreover, PSI Quantum photonic integrated circuits are fabricated using DUV lithography technology, which is usually not available for research groups.

In its turn, our paper serves as a tutorial on finding the best repeatable wafer-scale compatible coupling strategy for the Si_3_N_4_ platform using e-beam lithography fabrication technology, which is usually used by universities and scientists. The entire Si_3_N_4_ waveguide fabrication process comprises waveguide fabrication and plasma dicing based on deep silicon etching, which provides optical quality chip edge forming. In this article a universal approach for taper design is presented, which was used to simulate several types of inverse tapers. The coupling losses per coupler for the UHNA-7 fiber/chip coupling are calculated to be ∼0.10 dB and measured to be ∼0.15 dB for 1550 nm per connection. For a standard fiber/chip, coupling losses are calculated to be ∼0.51 dB and measured to be ∼1.50 dB for 1550 nm per facet. Further we describe in details how these results were achieved.

## 2. Simulation

### 2.1. Inverse Taper Simulation

**Figure 1:**
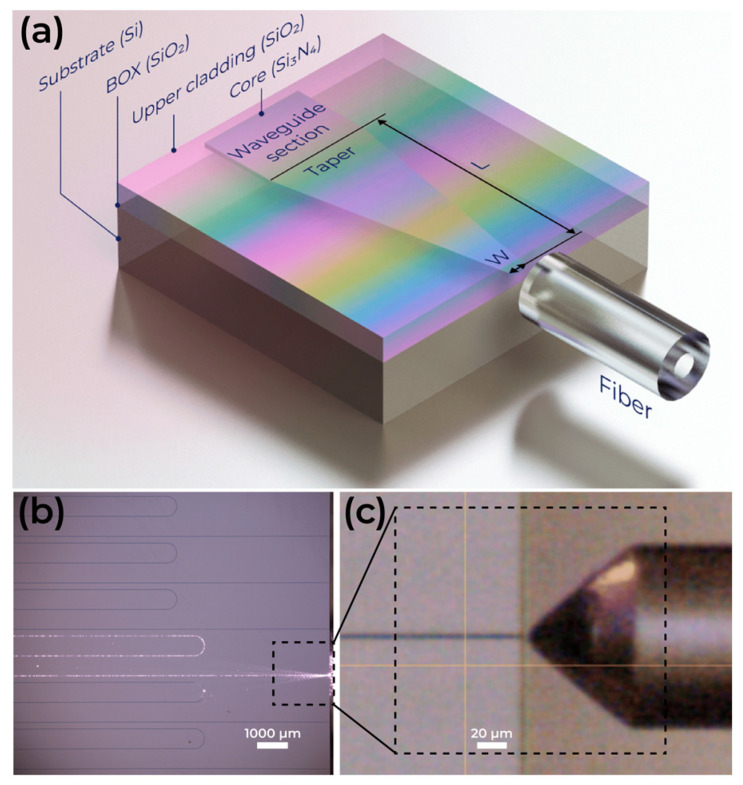
(**a**) Three-dimensional visualization of fiber coupled to taper. (**b**) Light propagation through the waveguide. (**c**) Coupling of lensed fiber with chip facet.

The proposed taper 3D model is shown in Figure 1a. Light propagation through the PIC after coupling via a lensed fiber is shown in Figure 1b,c.

**Figure 2:**
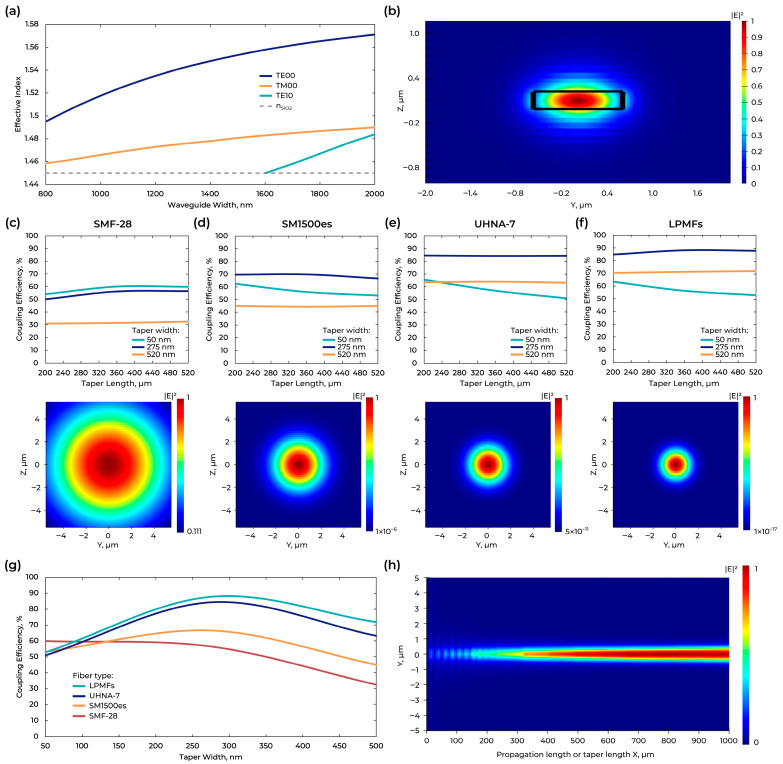
(**a**) Calculation of Si_3_N_4_ effective index of modes excited in a waveguide as a function of waveguide width. (**b**) Fundamental mode excited in the waveguide with 220 × 1200 nm cross-section. ((**c**–**f**), **top**) Calculation of the taper length at different widths for different fiber types with wavelength 1550 nm. ((**c**–**f**), **bottom**) Optical mode field distribution at the light-source facet with different fiber types. (**g**) Calculation of the taper width with fixed length of taper (360 µm) for different fiber types with wavelength 1550 nm. (**h**) Mode propagation in the on-chip taper under lensed-fiber excitation.

Modeling started with the waveguide geometry determination to ensure fundamental mode excitation (Figure 2a). Modeling was carried out in Ansys Lumerical finite-difference eigenmode (FDE), based on which a waveguide width of 1200 nm was chosen. The fundamental mode excited in the waveguide with 220 × 1200 nm cross-section is shown in Figure 2b.

To determine the optimal taper geometry, modeling was performed in Ansys Lumerical finite-difference time-domain (FDTD) based on the numerical solution of Maxwell’s equations. The length of the tapers varied from 200 µm to 520 µm. In its turn, the width at the beginning of the taper varied from 50 to 500 nm and was increased to the waveguide width value of 1200 nm. The smallest feature size, which we used in the modeling, is determined by the technological capabilities of electron lithography-based fabrication technology. The graphs (Figure 2c–f (top)) show the dependence obtained during the simulation for the different optical fibers. In this study, the taper length was chosen to be 360 μm to ensure low losses and a relatively small device footprint. To standardize the technological process, a taper geometry (width 280 nm, length 360 µm) was chosen to provide high coupling efficiency for all types of optical fibers (Figure 2g). Figure 2c–f (bottom), reports the optical mode field distributions at the light-source facet for representative fiber types (SMF-28, SM1500es, UHNA-7, and a Lensed fiber). In all field maps in Figure 2c–f (bottom), only the fundamental mode (TE_0_) at the light-source facet is shown. In Figure 2h, the simulated map shows the mode propagation inside the on-chip taper. The excitation is the focused output of a lensed fiber, represented as a Gaussian source at the chip facet.

### 2.2. Coupling Efficiency Improvement

**Figure 3:**
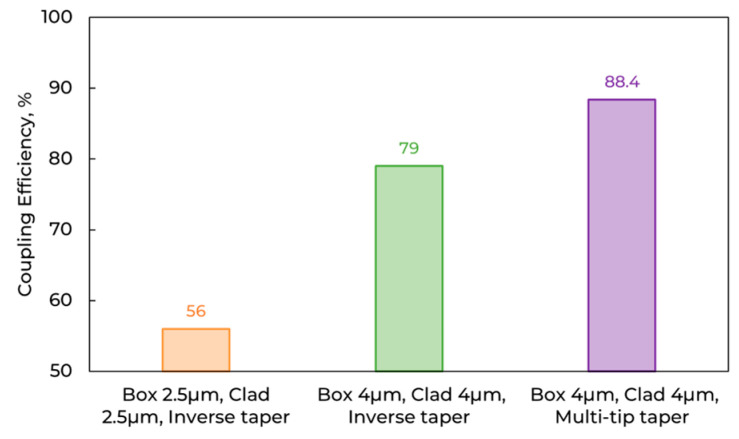
Simulated coupling efficiency versus SiO_2_ thickness and taper type.

Another approach to improve coupling efficiency is to use thicker cladding and a thicker bottom SiO_2_ layer, which mitigate vertical mode mismatch and reflections from the Si, respectively. Compared designs in Figure 3: inverse taper (width 280 nm; length 360 µm; BOX + Clad: 2.5 + 2.5; 4 + 4 µm) and multi-tip taper (tip 220 µm; start 100 nm; connection 5 µm; taper 40 µm; BOX + Clad: 4 + 4 µm) under SMF-28 excitation.

### 2.3. Multi-Tip Taper Simulation

One of the possible ways to reduce coupling losses is to use a multi-tip taper. Improving the geometry of the edge couplers by increasing the tip number often makes improvements to the coupling performance between optical fiber and optical channel on chip [50].

**Figure 4:**
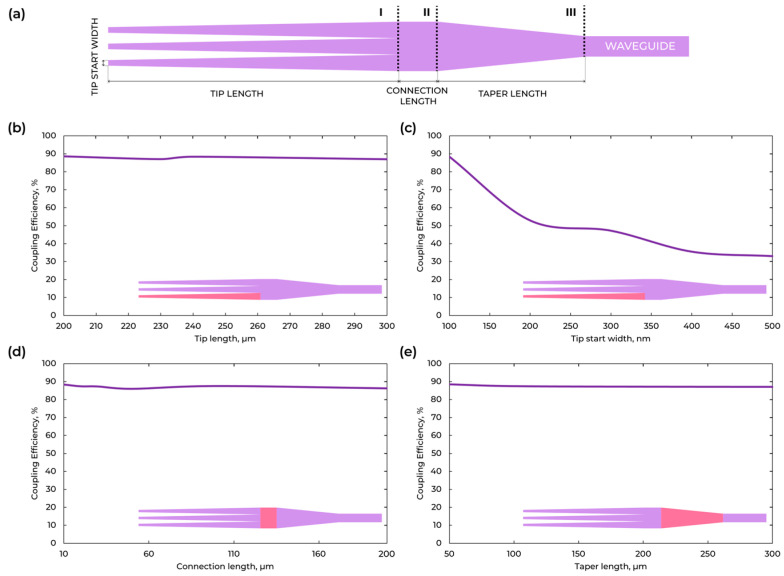
(**a**) Visualization of multi-tip taper parts. (**b**) Calculation of the tip length for multi-tip taper. (**c**) Calculation of the tip start width for multi-tip taper. (**d**) Calculation of the connection length for multi-tip taper. (**e**) Calculation of the taper length for multi-tip taper.

For example, for SMF-28 fiber mode, the field diameter is 10 μm, so a wide taper will allow the capture of more optical power from the fiber. And the multi-tip (trident-shape) taper will reduce the mismatch of the effective indices of the fiber and of the chip waveguide. Here we propose the multi-taper design for the SMF-28 fiber. Its design consists of three sections (Figure 4): a trident, a transition region, and an adiabatic taper. The tip length, tip start width, connection length, and taper length with the highest coupling efficiency are calculated by Ansys Lumerical FDTD. The results are shown in Figure 4. The chosen geometry of multi-taper has the following parameters: tip length = 220 µm, tip start width = 100 nm, connection length = 5 µm, and taper length = 40 µm. The calculated coupling losses with such multi-taper for SMF-28 fiber were improved from 2.52 to 0.51 dB.

**Figure 5:**
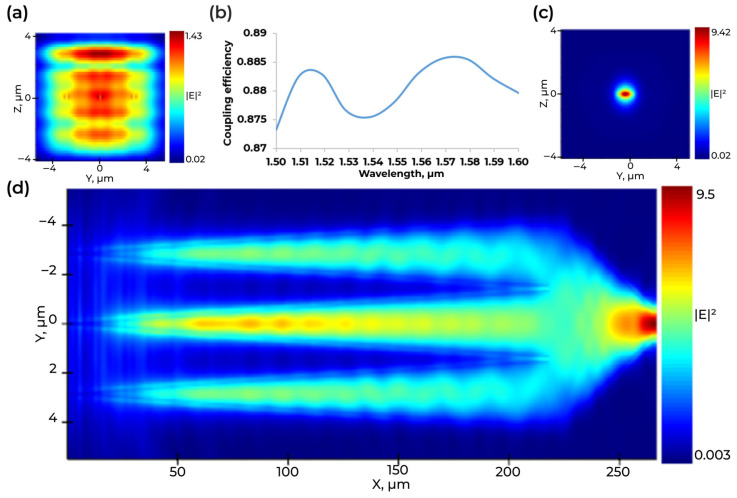
Simulation and spectral performance of the multi-tip taper. (**a**) Optical mode field distribution at the start of the multi-tip taper. (**b**) Wavelength-dependent coupling efficiency in the 1500–1600 nm range. (**c**) Optical mode field distribution at the start of the waveguide after the taper end. (**d**) Optical mode field transition along the longitudinal direction of the multi-tip taper.

We additionally present the optical mode field distribution at the start of the multi-tip taper (Figure 5a) and at start of the waveguide (Figure 5c), together with the field transition along the propagation direction of the multi-tip taper (Figure 5d). The longitudinal map (Figure 5d) shows a smooth, adiabatic evolution without standing-wave features, indicating robust recombination of the three tips with low phase sensitivity. The wavelength-dependent coupling efficiency within 1500–1600 nm (Figure 5b) remains in the 87–88.6% range. The small variation evidences broadband and spectrally stable performance of the multi-tip taper.

## 3. Fabrication

### Edge Facet

To provide high taper coupling efficiency, the PIC dies are typically polished to obtain high optical-grade quality edges [51]. Although this process works well with small chip sizes, its scaling up to wafer level is impractical. There is also used the process of silicon dioxide thick layers wet etching, but it is anisotropic, which negatively affects the quality of the optical facets [52]. Other solutions for PICs optical facets fabrication need to be investigated. A promising technology is the reactive plasma dicing [53]. In this work, a 4-inch (100 mm) Si wafer was used, and the chips were diced into 25 × 25 mm dies.

**Figure 6:**
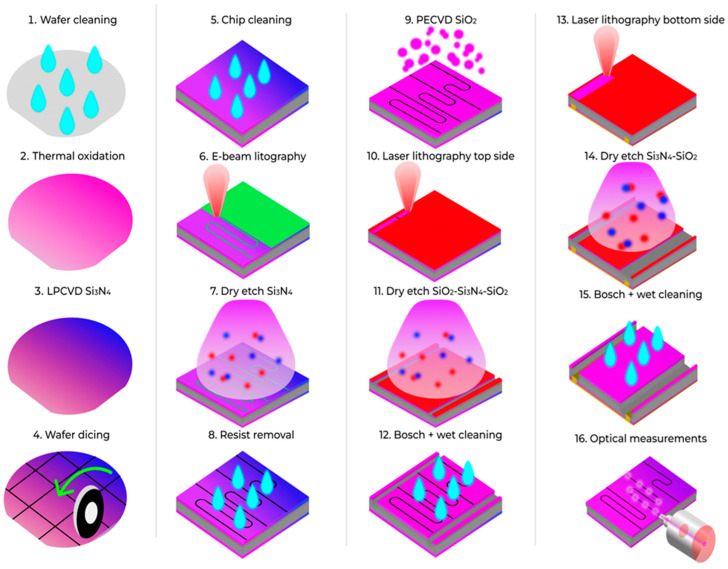
Si_3_N_4_ PICs with edge coupling fabrication process.

Current study continues the previous research dedicated to the near-infrared wavelength single-mode Si_3_N_4_ submicron waveguide platform (220 × 550 nm) [54] and presents a plasma-based process for optical-grade edge facets fabrication. The fabrication process of low-loss silicon nitride photonic integrated circuits with fiber coupling through edge couplers is shown in Figure 6. The presented technology is used to dice a chip that consists of a 220 nm-thick stoichiometric low-pressure chemical vapor Si_3_N_4_ layer grown on the 525-μm silicon substrate oxidized to the 2.5-μm oxide thickness and covered with 2.5-μm oxide. A standard sequence of basic operations is involved for fabrication (Figure 6). First, the waveguide structures and alignment marks were patterned using electron-beam lithography with ma-N 2403 resist and multipass exposure. The pattern is then transferred to the Si_3_N_4_ device layer by reactive ion plasma etching (RIE) [55] with subsequent resist removal step in the N-methyl-2-pyrrolidone (NMP) and Si_3_N_4_ active layer cleaning procedure. Afterwards, the PECVD cover oxide was deposited and furnace annealing was performed. After nanotopology patterning the chip is diced using the deep reactive ion etching (DRIE) of the SiO_2_–Si_3_N_4_–SiO_2_ layer and Bosch process of the Si layer. The dicing process consists of the following stages: surface preparation (O_2_-plasma activation), photoresist spin coating (SPR220), laser lithography, photoresist development (MF-24A), SiO_2_–Si_3_N_4_–SiO_2_ thin-film stack etching using fluorine gases, silicon etching by the Bosch process, and photoresist removal by soaking in acetone followed by an IPA rinse [55].

**Figure 7:**
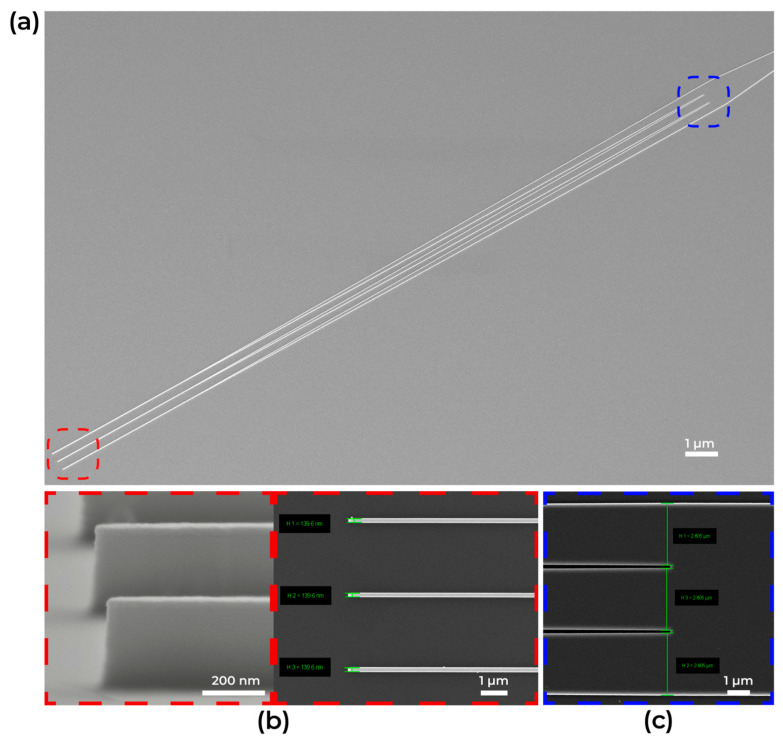
(**a**) SEM image of multi-tip taper. ((**b**), **red box**) SEM image of multi-tip taper start tips. ((**c**), **blue box**) SEM image of gap between multi-tip taper end tips.

In the multi-tip taper, the most critical electron-beam lithography dimensions are the tip width and the taper gaps, targeted at 100 nm. Following process development, scanning electron microscopy (SEM) confirmed a gap of 105 nm and a tip width of 140 nm (Figure 7).

**Figure 8:**
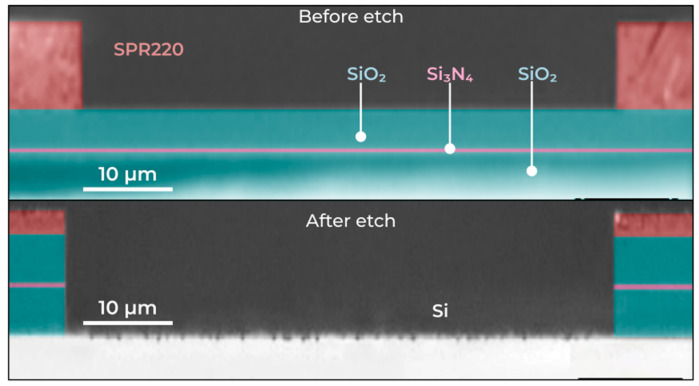
Optical image of angle profile after laser lithography (**top**) and after dry etch process (**bottom**).

To fabricate a photonic integrated circuit with thick oxide layers by deep etching, it is necessary to use a thicker resist layer. The processes of 9.5 μm-thick resist spin-coating and its laser lithography were developed. The 89.0° resist sidewall angle corresponded to the following lithography regime: multipass-technology, 60 mW laser power, −25% focus. Figure 8 shows the resist profile angle after the laser lithography step and the profile angle of the optical layers after the etching steps.

## 4. Characterization

### 4.1. Cut-Back Measurement

To measure the I/O efficiency, we used an automated assembly system equipped with a 12-axis alignment drive (5 nm resolution), a wavelength-stabilized 1550 nm laser source and an optical power meter. This system made it possible to measure internal optical losses and coupling efficiency using the output power values of structures of various lengths.

**Figure 9:**
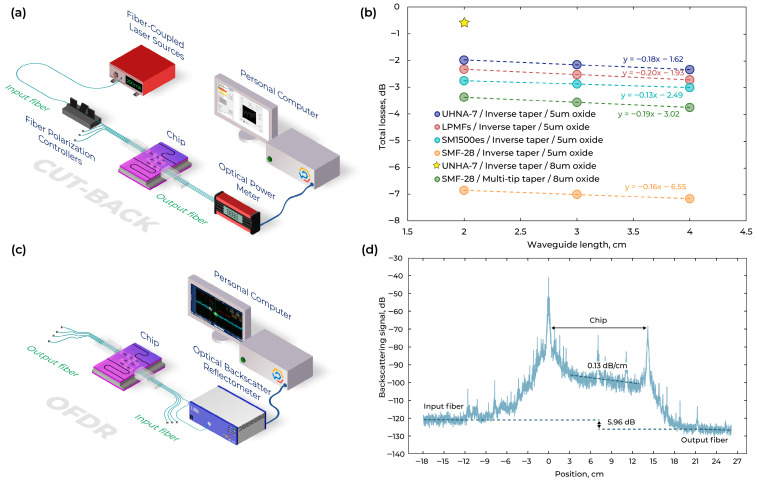
(**a**) Schematic of the measurement setup for cut-back characterization. (**b**) Propagation losses measured by the cut-back, calculated by y = k × x + b where k = propagation losses and b = coupling losses for wavelength 1550 nm. (**c**) Schematic of the measurement setup for OFDR characterization. (**d**) OFDR characterization along the waveguide. The dashed line shows the linear fit of the waveguide reflections.

The cut-back propagation loss analysis [56,57] in the fabricated Si_3_N_4_ waveguides was performed for specially designed PIC chips. Propagation losses were measured for the test structure with three different lengths (2, 3, and 4 cm). Measurement setup is shown in Figure 9a. Figure 9b presents the measured losses for different fiber types and different SiO_2_ layers thickness. The light was coupled out from the polished fiber array unit (FAU) through PIC to the power detector.

The lowest coupling losses for the inverse taper of 0.81 dB (violet line in Figure 9b, 0.81 = 1.62/2 dB) were observed for the UHNA-7 fiber. For the device with increased cladding oxide thickness (Figure 9b, star), the measured total insertion loss was TL = 0.66 dB at L = 2 cm, and the propagation loss from the cut-back fit was PL = 0.18 dB/cm. The total loss of a straight device is given by

(1) TL=2·CL+PL·L

where CL is the coupling loss per facet. Substituting the measured values gives

(2) CL=(TL−PL·L)/2=(0.66−0.18·2)/2=0.15 dB per facet

### 4.2. OFDR Measurements

Cut-back measurements were cross-checked using a reflectometry technique. Reflectometry is widely used in fiber optics to probe the local reflectivity of waveguides and devices with respect to propagation distance [58]. Figure 9c shows schematic of the measurement setup for optical frequency domain reflectometry (OFDR) characterization. With a point-to-point resolution of about 10 µm and detection sensitivity of −130 dB over 30 m of propagation, coherent OFDR is a particularly useful technique in characterizing waveguides and devices at the planar scale [59,60]. In the OFDR, a continuous wave laser source is scanned over several terahertz in frequency or, equivalently, several tens of nanometers in wavelength. A larger scan range improves the measurement spatial resolution according to the following expression:

(3) $$D_{min}≅\frac{c}{2n_{g}\left|f_{start}-f_{end}\right|}=\frac{\lambda_{start}\lambda_{end}}{2n_{g}\left|\lambda_{start}-\lambda_{end}\right|}$$

where *D_min_* is the minimum distance between the two data points, c is the speed of light, *n_g_* is the group index, *f_start_* and *λ_start_* are the source frequency and wavelength at the scanning start, respectively, and *f_end_* and *λ_end_* are the source frequency and wavelength at the scanning end [32]. However, all parameters derived from the spatial domain data are then averaged over the measurement scan spectral range. If the parameter spectral dependence is set, the rectangular window function can be applied to data in the spectral domain to narrow the included spectral range. This window could be moved across the measurement’s full spectral range, making it possible to extract the parameter at each window position and obtain the parameter spectral dependence from a single OFDR scan. Because narrowing data in the frequency domain decreases the measurement spatial resolution according to Equation (3), a tradeoff appears between the spectral averaging, which could distort the actual spectral dependence and the measurement accuracy. In this study, an FDTD window width of 10 nm is used to extract the spectrally dependent measurements of the coupling loss propagation loss.

Figure 9d shows the OFDR data from two UHNA-7 fibers coupled to waveguides (10 cm long). The data was not filtered in the spectral domain, and a moving average filter with 100-datapoint or ~1 mm window size was applied in the spatial domain to reduce the backscatter amplitude deviation. Before OFDR scanning, fiber-to-chip coupling was maximized using an OFDR source laser and an optical power meter. In Figure 9d, the horizontal dashed lines indicate the total loss across the device (input coupling + waveguide + output coupling), while the dashed sloped line represents the linear fit from which the waveguide propagation loss (dB/cm) is obtained. The difference between these two levels of 5.96 dB includes the total return loss between the two fibers, or,

(4) $${RL}_{dB}^{total}={2IL}_{dB}^{total}=2\left({IL}_{dB}^{fiber-to-chip}+{IL}_{dB}^{propagation}+{IL}_{dB}^{fiber-to-chip}\right)$$

where ${RL}_{dB}^{total}$ is the total return loss in dB, ${IL}_{dB}^{total}$ is the total insertion loss, ${IL}_{dB}^{fiber-to-chip}$ is the fiber-to-chip insertion loss per facet, and ${IL}_{dB}^{propagation}$ is the total propagation insertion loss.

The measured data is linearly fitted to find propagation losses (Figure 9d). Based on OFDR characterization, the propagation insertion loss is 0.13 dB/cm and fiber-to-chip insertion loss per facet is 0.84 dB. That corresponds well to the results obtained with cut-back measurements: coupling losses of 0.81 dB and propagation losses of 0.18 dB/cm (violet line in Figure 9b).

## 5. Discussion

This study presents a universal strategy for repeatable wafer-scale PIC low-loss edge coupling. Here we provide a tutorial on the design, manufacturing, and characterization of lithographically defined optical coupling facets using an ICP dry etching technique. We also designed, fabricated, and experimentally validated inverse and multi-tip taper for UHNA-7 and SMF-28 fibers.

The coupling geometries were optimized using numerical simulations based on FDE and FDTD methods. The waveguide cross-section was first selected to ensure robust single-mode operation, after which the taper length and tip width were varied to achieve adiabatic mode transformation for different fiber types. The simulations indicate that a taper length of approximately 360 μm offers a favorable trade-off between coupling efficiency and device footprint, while remaining compatible with electron-beam lithography constraints. The optimized design offers calculated coupling losses for UHNA-7 fiber at the level of ∼0.10 dB and for SMF-28 fiber at the level of ∼0.50 dB be for 1550 nm per facet. To address the strong mode mismatch for standard SMF-28 fibers, a multi-tip (trident-shaped) taper was introduced, enabling a smoother modal transition by redistributing the optical field among several narrow tips. The chosen geometry of multi-taper has the following parameters: tip length = 220 µm, tip start width = 100 nm, connection length = 5 µm, and taper length = 40 µm. The optimized multi-tip design exhibits a marked reduction in calculated coupling losses and maintains stable performance over the 1500–1600 nm wavelength range and calculated coupling losses with such multi-taper for SMF-28 fiber were improved from 2.52 to 0.51 dB.

The proposed coupling structures were fabricated using a wafer-scale Si_3_N_4_ photonic process incorporating DRIE-based plasma dicing. This approach enables the formation of optical-grade facets without mechanical polishing, thereby improving reproducibility and scalability. Inverse tapers and multi-tip structures were defined by electron-beam lithography, with critical feature sizes approaching 100 nm. Scanning electron microscopy confirmed good agreement between the designed and fabricated dimensions. Fabrication process optimization allowed us to obtain the 89° optical-grade facet, which is essential for minimizing scattering and reflection losses during fiber-to-chip coupling.

Device performance was characterized using cut-back measurements and OFDR. OFDR measurements independently verified the extracted coupling and propagation losses and showed good consistency with the cut-back analysis. When interfacing with SMF-28 fibers, the multi-tip taper demonstrated a measured coupling loss of approximately 1.5 dB per facet, confirming the effectiveness of the proposed design in mitigating mode mismatch. The best coupling losses of 0.15 dB were achieved for the UHNA-7 fiber with single-mode Si_3_N_4_ waveguide (220 × 1200 nm), that agrees well with the simulation results.

## Figures and Tables

**Table 1:** Si_3_N_4_ taper coupling losses overview.

Reference	Taper Length / Width	Coupling Efficiency, Measured (Calculated)	3 dB Alignment Tolerance, μm Measured (Calculated)		Fiber Type (Mode Size, μm)	Waveguide Cross-Section/Technology
		TE Mode Losses, dB	Horizontal	Vertical		
[42]	500 μm / 300 nm	0.17 (0.087)	±3.8 (±3.6)	±3.6 (±3.5)	UHNA-3 (4.1 ± 0.3)	100 × 900 nm/a single step litho
[35]	500 μm / 180 nm	2.00	-	-	LPMFs (2.5 ± 0.3)	400 × 700 nm/polished with diamond films
[39]	45 μm / 750 nm	1.47 (0.58)	– (±1.0)	– (±1.0)	UHNA-3 (4.1 ± 0.3)	300 × 1000 nm/shadow mask
[48]	76 μm / 200 nm	0.36 (0.29)	±3.5	±3.3	UHNA-3 (4.1 ± 0.3)	500 × 2000 nm/a single step litho
[46]	100 μm / ~300 nm	0.70 (0.70–0.80)	-	-	LPMFs (3.0)	400 nm thick/ trident edge coupler, single etch step, no substrate removal 8 μm oxide thickness
[49]	-	0.127 ± 0.018	-	-	SMF-28	-
	-	0.052 ± 0.012	-	-	UHNA-4	
Current work	360 μm/ 275 nm	3.28 (2.52)	±2.8 (±3.2)	±2.4 (±2.9)	SMF-28 (10.5 ± 0.5)	220 × 1200 nm/double step litho, 5 μm oxide thickness
		0.97 (1.55)	±1.4 (±2.0)	±1.1 (±1.5)	SM1500es (4 ± 0.5)	
		1.25 (0.55)	±0.9 (±1.5)	±0.7 (±1.2)	LPMFs (2.5 ± 0.3)	
		0.81 (0.75)	±1.2 (±1.7)	±0.8 (±1.3)	UHNA-7 (3.2 ± 0.3)	
	280 μm/500 nm	0.15 (0.10)	– (±1.2)	– (±0.9)	UHNA-7 (3.2 ± 0.3)	20 × 1200 nm/double step litho, 8 μm oxide thickness
Current work Multi-tip taper	265 μm/ 8.55 μm	1.50 (0.51)	– (±5)	– (± 3)	SMF-28 (10.5 ± 0.5)	220 × 1200 nm/double step litho, 8 μm oxide thickness

## Data Availability

Data supporting the findings are available within the article and from the corresponding author upon reasonable request.

## Abbreviations

The following abbreviations are used in this manuscript:

UHNA	Ultra-High Numerical Aperture
PIC	Photonic integrated circuits
QKD	Quantum key distribution
SOI	Silicon-on-insulator
MPW	Multi-Project Wafer
DUV	Deep ultraviolet
FDE	Finite-difference eigenmode
FDTD	Finite-difference time-domain
FAU	Fiber array unit
DRIE	Deep reactive ion plasma etching
RIE	Reactive ion plasma etching
ICP	Inductively Coupled Plasma
OFDR	Optical frequency domain reflectometry
SMF	Single-Mode Fiber
